# Analysis of computational tumor-infiltrating lymphocytes in breast cancer from the results of the TIGER challenge

**DOI:** 10.1038/s41467-026-72956-x

**Published:** 2026-05-15

**Authors:** Mart van Rijthoven, Witali Aswolinskiy, Leslie Tessier, Roberto Salgado, Jeroen van der Laak, Francesco Ciompi, TIGER consortium, Witali Aswolinskiy, Leslie Tessier, Roberto Salgado, Francesco Ciompi, Mart van Rijthoven, Jeroen van der Laak, Maschenka Balkenhol, Joep M. A. Bogaerts, Damien Drubay, Laura Comerma Blesa, Dieter Peeters, Elisabeth Specht Stovgaard, Anne-Vibeke Lænkholm, Harry Haynes, Ligia Craciun, Denis Larsimont, Mohamed T. Amgad, Lee AD Cooper, Cyril de Kock, Valerie Dechering, Johannes Lotz, Nick Weiss, Mieke van Bockstal, Christine Galant, Esther Lips, Hugo M. Horlings, Jelle Wesseling, Lennart Mulder, Sandra van den Belt, Karsten Weber, Paul Jank, Carsten Denkert, Enrico Munari, Giuseppe Bogina, Chris Russ, Alex Lemm, Sherene Loi, Julia Dixon-Douglas, Stefan Michiels, Rogier Donders, Scott Maurits, Miriam Groeneveld, Anne Mickan, James Meakin, Bram van Ginneken, Heikki Joensuu, Ming Fan, Daehong Lee, Jaehyung Ye, Kangwon Byun, Jeongyeol Kim, Shuoyu Xu, Zheng Ji, Feng Xie, Jinbo Kuang, Xulin Chen, Liliang Chen, Arian Arab, Weijie Chen, Victor Garcia, Nicholas Petrick, Brandon Gallas, Anna Maria Tsakiroglou, Richard Byers, Martin Fergie, Vishwesh Ramanathan, Anne L. Martel, Adam Shephard, Shan E. Ahmed Raza, Mostafa Jahanifar, Nasir M. Rajpoot, Sungduk Cho, Dong-Hee Kim, Hyungjoon Jang, Chanmin Park, Kyungdoc Kim

**Affiliations:** 1 Radboud University Medical Center, Nijmegen, The Netherlands; 2 Division of Cancer Research, Peter MacCallum Cancer Centre, Melbourne, VIC Australia; 3 Department of Pathology, ZAS Hospitals, Antwerp, Belgium; 4 Center for Medical Image Science and Visualization, Linköping University, Linköping, Sweden; 5 Canisius Wilhelmina Ziekenhuis, Nijmegen, The Netherlands; 6 Gustave Roussy, Université Paris-Saclay, Villejuif, France; 7 Hospital del Mar, Barcelona, Spain; 8 Universitair Ziekenhuis Antwerpen, Antwerpen, Belgium; 9 Department of Pathology, Herlev and Gentofte Hospital, Herlev, Denmark; 10 Department of Clinical Medicine, University of Copenhagen, Copenhagen, Denmark; 11 Zealand University Hospital, Køge, Denmark; 12 Great Western Hospitals NHS Foundation Trust, Swindon, UK; 13 Pathology, Institut Jules Bordet, Université Libre de Bruxelles (ULB), Brussels, Belgium; 14 Department of Pathology, Northwestern University Feinberg School of Medicine, Chicago, IL USA; 15 Fraunhofer Institute for Digital Medicine MEVIS, Lübeck, Germany; 16 Department of Pathology, Cliniques Universitaires Saint-Luc, Brussels, Belgium; 17 Pôle MORF, Institut de Recherche Expérimentale et Clinique (IREC), Université catholique de Louvain, Brussels, Belgium; 18 The Netherlands Cancer Institute (NKI), Amsterdam, The Netherlands; 19 GBG German Breast Group, Neu-Isenburg, Germany; 20 Institute of Pathology, Philipps University Marburg and Marburg University Hospital (UKGM), UCT Frankfurt-Marburg, Marburg, Germany; 21 Department of Pathology and Diagnostics, University and Hospital Trust of Verona, Verona, Italy; 22 IRCCS Sacro Cuore Don Calabria Hospital, Negrar di Valpolicella, Verona, Italy; 23 Amazon Web Services EMEA SARL (AWS Europe), Luxembourg, Luxembourg; 24 The Sir Peter MacCallum Department of Medical Oncology, The University of Melbourne, Parkville, VIC Australia; 25 Department of Oncology, Helsinki University Hospital and University of Helsinki, Helsinki, Finland; 26 Aivis Inc, Seoul, Republic of Korea; 27 Department of Pathology, Korea University Guro Hospital, Korea University College of Medicine, Seoul, Korea; 28 Bio-totem Pte Ltd, Suzhou, PR China; 29 Cells Vision (Guangzhou) Medical Technology Inc, Guangzhou, China; 30 Division of Imaging, Diagnostics and Software Reliability, U.S. Food and Drug Administration, CDRH, OSEL, Silver Spring, MD USA; 31 Spotlight Pathology Ltd, Manchester, UK; 32 Department of Medical Biophysics, University of Toronto, Toronto, ON Canada; 33 Physical Sciences, Sunnybrook Research Institute, Toronto, ON Canada; 34 Tissue Image Analytics Centre, Department of Computer Science, University of Warwick, Coventry, UK; 35 VUNO Inc, Seoul, South Korea

**Keywords:** Breast cancer, Lymphocytes, Computational science

## Abstract

Tumor-infiltrating lymphocytes (TILs) is a recognized prognostic biomarker in breast cancer. However, poor interobserver agreement and limited reproducibility highlight the need for computational approaches. Despite advances, adoption of computational models has been hindered by lack of standardized methods and robust benchmarks. To address this, we launched TIGER, an international competition to build open-source computational TILs (cTILs) models. Here, we present a multi-centric analysis of cTILs methods on resections and biopsies from 3,708 human epidermal growth factor receptor 2-positive (HER2+) or triple-negative breast cancers (TNBC) from clinical practice and phase 3 trials. We report benchmarks on image analysis performance, show strong agreement of cTILs with pathologists, and demonstrate positive association of cTILs with neoadjuvant therapy response in HER2+, superior to visually scored TILs. We also show that cTILs add independent prognostic information to clinical variables in TNBC resections. Data, methods and benchmarks are publicly available: https://tiger.grand-challenge.org/.

## Abstract (web summary)

Comprehensive benchmarking of computational methods for the prognostic significance of tumour-infiltrating lymphocytes (TILs) in breast cancer remains elusive. Here, the authors perform multi-centric validation and evaluation of multiple cTILs methods in a large cohort of triple negative and HER2+ breast cancer patients.

## Introduction

Since the beginning of the 20th century, clinicians observed that breast cancer (BC) patients with higher levels of lymphocytic infiltration in tumors, now defined as tumor-infiltrating lymphocytes (TILs), often had a better prognosis compared to patients with lower levels^1^. Over the years, high TILs density has also been shown to be associated with better responses to immunotherapy and chemotherapy. However, the lack of a formal and universally shared method to quantify the TILs hampered for many years the translation of those clinical observations into a biomarker, until recently.

In 2015, the International Immuno-oncology Biomarker Working Group (also known as the TILs WG) proposed recommendations^2^ on evaluating and measuring stromal TILs (sTILs) in hematoxylin and eosin (H&E) stained histopathology slides of BC, de facto reviving interest in TILs and their role as a biomarker in immuno-oncology^3^. Using these recommendations, research initially focused on measuring inter-observer variability in visual TILs quantification. A study conducted by the TILs WG^4^ found very good concordance rates across several clinically relevant cut-points, but also reported inter-observer disagreement, for example, in the presence of heterogeneous infiltration of immune cells^5^. A more recent study reported substantial interobserver disagreement when TILs are scored on core-needle biopsies^6^. A large study on a pooled individual patient analysis from phase 3 clinical trials^7^ showed the prognostic value of sTILs as a biomarker for cancer recurrence and patient survival in early-stage triple-negative breast cancer (TNBC). Studies^8,9^ have also shown the correlation of sTILs with pathological complete response (pCR) following neoadjuvant chemotherapy (NACT) in early-stage TNBC and human epidermal growth factor receptor 2-positive (HER2+ ) breast cancers, as well as correlation with cancer recurrence and survival, regardless of the hormone receptor (HR) status. Recent initiatives are investigating the impact of TILs as a biomarker to forgo chemotherapy in TNBC cases^10^.

Aiming at reducing inter-observer variability and complementing pathologists where TIL-evaluation can be difficult, researchers have focused on artificial intelligence (AI) models^11^ to automate computational TILs (cTILs) quantification in digital pathology whole-slide images (WSI). Most presented models rely on deep learning to classify small image regions (i.e., patches)^12–14^, analyze tissue morphology (i.e., detect cells and segment tissue)^8,15–18^, also using open-source tools^19,20^, or end-to-end learning from raw data^21^. The bulk of work on cTILs has focused on breast cancer^5,9,16,18,19,21–25^, but similar solutions have also been proposed for other cancer types such as melanoma^20^, gastric^12^, head and neck^17^, lung^26,27^, oropharyngeal^15^ and testicular cancer^28^. In colorectal cancer, the Immunoscore^29^ has been proposed to quantify immune response based on the immunohistochemical (IHC) analysis of CD8- and CD3-positive T lymphocytes at the central part and invasive front of the tumor. Partly inspired by the Immunoscore approach, AI-based quantification of peritumoral lymphocytes in H&E-stained slides has been found to hold prognostic value in TNBC^30^. However, so far no computational method has been considered to have sufficient level of clinical validity to be usable in daily practice settings. This is mostly due to a) the lack of consensus or recommendations on how to design computational scoring methods, and b) the lack of large-scale studies to comprehensively benchmark cTILs across multiple clinical end-points, including correlation with visual assessment in both biopsies and surgical resections, and clinical validation on large scale clinical trials. Given these challenges, we sought to systematically benchmark cTILs models through an international effort: the Tumor InfiltratinG lymphocytes in breast cancER (TIGER) challenge.

**Fig. 1:**
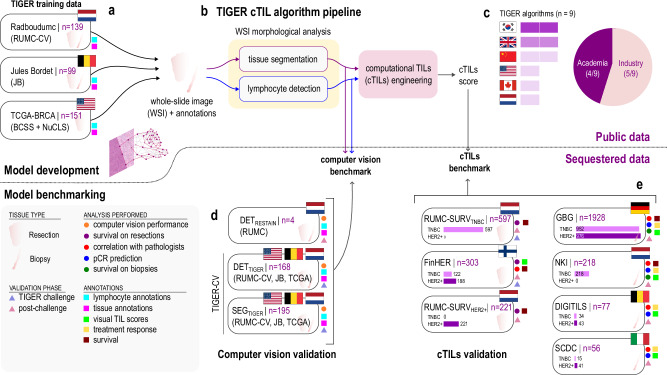
Overview of cTILs algorithm development and benchmarking. **a** Publicly available multi-centric training data, containing WSIs with corresponding manual annotations released as part of the TIGER challenge to train the morphological analysis component of cTILs models. **b** Schematic overview of the components of cTILs models developed in TIGER. Manually annotated training WSIs were used to build computer models for tissue segmentation and lymphocyte detection. The output of these models was used to engineer a cTILs score. Both models for morphological analysis and the cTILs score were benchmarked in this study. **c** From the final phase of TIGER, we obtained a total of nine AI models from six different countries, both from academia (*n* = 4) and industry (*n* = 5). **d** Sequestered data used to benchmark the computer vision performance (i.e., lymphocyte detection and tissue segmentation) of the morphological analysis module. We used the multi-centric TIGER-CV dataset (the test set of the final phase in the computer vision task of TIGER, consisting of DET_TIGER_ for cell detection and SEG_TIGER_ for tissue segmentation) and DET_RESTAIN_ for cell detection, with an immunohistochemistry-based reference standard. **e** Sequestered data used to benchmark several aspects of cTIL): (i) prognostic value on surgical resections, (ii) prognostic value on biopsies, (iii) correlation with pathologists’ visual sTILs, (iv) prediction of pCR in neoadjuvant chemotherapy treatment. We included both HER2+ and TNBC in all sets. See Table 2 for an overview on data acquisition processes. Abbreviations: cTILs: computational tumor-infiltrating lymphocytes; HER2+ : human epidermal growth factor receptor 2-positive; TNBC: triple-negative breast cancer; TCGA: The Cancer Genome Atlas; DET: detection; SEG: segmentation; WSI: whole-slide image; pCR: pathological complete response. Legend: TIGER challenge validation phase indicates analyses performed during the challenge and publicly available on Grand Challenge leaderboards. Post-challenge validation phase indicated analysis solely performed after the TIGER challenge for this study.

The TIGER challenge is a two-phase Grand Challenge (https://tiger.grand-challenge.org/) (Supplementary section TIGER challenge design), focusing on the most aggressive types of BC, namely TNBC and HER2+ breast cancer. With TIGER, we involved the scientific community to develop AI models that analyze tissue morphology in digital pathology whole-slide images and generate automated cTILs scores. We publicly released a multi-centric dataset of images and manual annotations to allow participants to train AI models (Fig. 1a), and a public evaluation procedure on the Grand Challenge^31^ (GC) platform. Via GC, we implemented a privacy-preserving benchmarking procedure, with model validation based on sequestered (i.e., not directly accessible by participants) whole-slide images. We used this approach to benchmark AI models both from a computer vision perspective (i.e., the automated identification of tumor and stroma tissue regions in images) and from a clinical perspective (i.e., the analysis of the prognostic value of cTILs). We also promoted an open and reproducible science approach by releasing training data, the code of baseline AI model and evaluation metrics, and required all participants to open source their final cTILs solution.

The output of the TIGER challenge is the foundation of the study presented in this paper. While retracing the main phases of the challenge, we extend them here with a comprehensive post-challenge analysis to benchmark additional aspects of cTILs models obtained from the final phase of TIGER. For this purpose, we establish a multicenter international dataset for cTILs validation, consisting of *n* = 3708 early-stage breast cancers (*n* = 1938 TNBC, *n* = 1770 HER2+ , see Methods section Materials) from clinical practice and phase 3 clinical trials, including digital pathology whole-slide images of both surgical resections and preoperative (core-needle) biopsies. First, for each cTILs algorithm we report results of the computer vision benchmark (Fig. 1d), measuring tissue segmentation and lymphocyte detection performance. For this, we use an internal validation dataset from the TIGER challenge enriched by a post-challenge dataset with reference standard based on lymphocyte-sensitive immunohistochemistry (Supplementary section Re-stained data). Second, we report results of the cTILs benchmark using a large post-challenge validation set (Fig. 1e, Table 2) that is completely independent from the TIGER training data. As part of the cTILs benchmark we measure (a) the correlation of cTILs with visual scoring of pathologists in both surgical resections and biopsies; (b) the association between cTILs and pCR in a NACT setting, using core-needle biopsies as input; and (c) the prognostic value of cTILs (assessed on both biopsies and surgical resections) in terms of disease-free interval (DFI) and overall survival (OS).

## Results

We validated all cTILs models that made valid submissions to the final phase for survival evaluation of the TIGER challenge, as well as the top-3 AI models for the final phase for the computer vision evaluation. This resulted in nine open-source models (Fig. 1c): aivis, biototem, cellsvision, didsr, radboud, spotlight, sri, tiager, vuno (see Supplementary section*TIGER challenge evaluation* and *Survival track* for more information about the TIGER challenge setup and the survival phase, and Supplementary section TIGER Models for descriptions of AI models).

The following applies to results reported in this study: aivis only made valid submissions to the computer vision track, therefore it was not included in the cTILs benchmark; the radboud algorithm was developed before TIGER and proposed by the challenge organizers as the baseline solution to improve upon within TIGER, it did not officially compete in the challenge and is reported here for completeness; sri only made submissions to the survival track, therefore it was not included in the computer vision benchmark.

We structured the presentation of our results in a stepwise manner. First, we report on the technical computer vision performance of each algorithm on tissue segmentation and lymphocyte detection. Second, we compare cTILs with pathologists’ visual assessments. Third, we evaluate associations of cTILs with clinical endpoints derived from the breast cancer treatment clinical pathway: prediction of response to neoadjuvant chemotherapy on biopsies followed by survival analyses on tissue from biopsies and surgical resections after adjuvant treatment.

### Computer vision benchmarks

We evaluated the performance of cTILs models to analyze the morphology of whole-slide images, consisting in (a) the segmentation (i.e., pixel classification) of tumor and stroma regions and (b) detection (i.e., prediction of location) of lymphocytes.

**Fig. 2:**
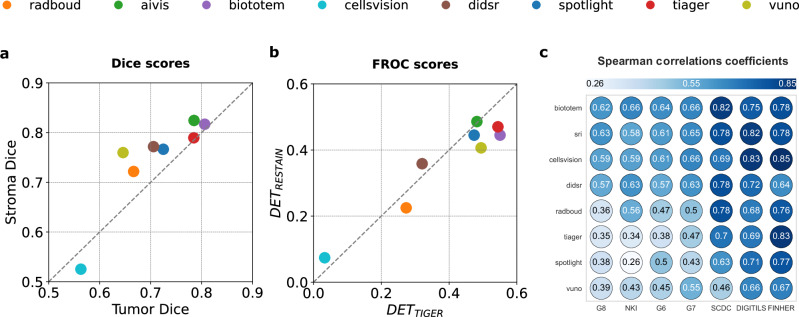
Results of TIGER models for computer vision performance and correlation with pathologists. **a** segmentation performance (*n* = 195 patients): scatter plot of Dice score for tumor versus stroma for each algorithm). **b** detection performance: scatter plot of FROC scores for each algorithm when tested on DET_TIGER_ (*n* = 168) versus DET_RESTAIN_ (*n* = 4). **c** Correlation with visual TILs: Spearman rank correlation coefficients for cTILs from each final TIGER algorithm with vTILs assessed by pathologists for each cohort; Sample sizes: G6 (*n* = 553), G7 (*n* = 617), G8 (*n* = 758), DIGITILS (*n* = 77), NKI (*n* = 218), SCDC (*n* = 56), FinHER (*n* = 303). The aivis algorithm did not produce a cTILs score and is excluded from this analysis. Each circle’s color and numerical value indicate the correlation coefficient. Rows and columns are sorted based on the average correlation per algorithm (rows) and per dataset (columns). Abbreviations. FROC: Free-Response Receiver Operating Characteristic; DET: detection; cTILs: computational tumor-infiltrating lymphocytes; vTILs: visual tumor-infiltrating lymphocytes (computed as a combination of visual TILs assessments by a group of pathologists). Source data are provided in the source data file linked to this paper.

For *segmentation*, we used the SEG_TIGER_ subset of TIGER-CV from the final phase of the computer vision track of TIGER, consisting of 286 regions of interest (ROIs), sampled from 195 H&E WSIs, with exhaustive manual annotations (i.e., all pixels annotated) of three tissue classes (tumor, stroma and other tissue). For each algorithm, we computed the Dice score, which measures the overlap between each segmented region and the corresponding manual annotations. We computed Dice for the tumor and the stroma classes over the whole set of ROIs. We found that all AI models segmented tumor equally well as stroma (Fig. 2a), with a slightly better performance on stroma segmentation (average Dice = 0.75) vs tumor segmentation (average Dice = 0.71). Detailed numerical values are shown in Supplementary table 6.

For *detection*, we used the DET_TIGER_ and DET_RESTAIN_ datasets. DET_TIGER_ was the set of manual lymphocyte annotations in TIGER-CV (Fig. 1d), consisting of 1879 ROIs taken from 168 H&E WSIs, with lymphocyte locations annotated on the slide by five pathologists, one pathologist per slide. DET_RESTAIN_ was built using four paired images of the same slides stained with H&E first and then re-stained with IHC (CD3 for T lymphocytes and CD79a for B lymphocytes). Manual annotations of lymphocyte locations were made on the IHC slide and transferred to the H&E image using an algorithm of image registration^32^ (i.e., image alignment). For each algorithm, we computed Free-Response Receiver Characteristics (FROC)^33^ curves and derived a FROC score by averaging sensitivities computed at 10, 20, 50, 100, 200 and 300 false positives per *mm*^2^. Results are depicted in Fig. 2b. FROC scores show that the AI models detect lymphocytes equally well on DET_TIGER_ and DET_RESTAIN_, although with a general trend to lower scores in DET_RESTAIN_ (range [0.07–0.49] in DET_RESTAIN_ vs. [0.03–0.55] in DET_TIGER_). Using DET_RESTAIN_ served both as a way to extend the benchmark including an objective reference standard, and as an indirect way to compare, although qualitatively, manual lymphocyte annotations used in TIGER with IHC-based annotations.

Overall, we found that performance rank of AI models is similar in segmentation and detection tasks resulting in biototem, tiager and aivis as the three top-performing AI models. For detailed descriptions of the datasets, annotations, evaluation metrics, and supplementary analyses, refer to Supplementary section Building Annotated Data for TIGER, Re-stained Data, TIGER Challenge Evaluation, and Methods*:* Computer Vision Performance, as well as Supplementary Table 4 for the final computer vision performance.

### Computational TILs benchmarks

We analyzed cTILs scores using several benchmarks, namely (a) the correlation of cTILs with visual TIL scores from pathologists, (b) the association of cTILs with response to neoadjuvant chemotherapy, (c) the prognostic value of cTILs considering disease-free interval (DFI) and overall survival (OS) as end points.

### cTILs show on average strong correlation with visual TILs

We analyzed the correlation between cTILs and visual scoring of stromal TILs (sTILs) assessed by pathologists following the TIL WG recommendations^2^. We used data from 2582 BC cases, including both biopsies and resections for which visual TIL scores were available. For biopsies, we used 2,279 WSIs from four cohorts (Fig. 1e): DIGITILS (*n* = 77), three cohorts from the GBG (*n* = 1928 in total) consisting of GeparSixto (G6, *n* = 553), GeparSepto (G7, *n* = 617), GeparOcto (G8, *n* = 758), NKI (*n* = 218) and SCDC (*n* = 56). For resections, we used a subset of *n* = 303 cases from FinHER (Method section *Data*). In all cohorts, visual TILs were scored by one or more pathologists (range [1–40]). Following Van Bockstal et al.^34^, we computed a median pathologist score (vTILs) as the slide-level median sTIL (Method section Combination of sTILs) and used it as a reference to compute the Spearman rank correlation coefficient (ρ) with cTILs. Figure 2c depicts an overview of the correlation values ranked per algorithm and per dataset (Supplementary Section: Scatter Correlations shows the trends of each pair of vTILs and cTILs).

We found that all cTILs showed on average strong correlation with vTILs (mean ρ value = 0.61, range [0.26–0.85]), with top-3 highest correlations for biototem, sri and cellsvision, and on average highest correlation on the FinHER dataset, which contains surgical resections. In some cases, weak correlation was found in G8 and NKI for radboud, tiager, spotlight and vuno. Interestingly, these methods mostly used a concave hull-based approach to define the region of the tumor bulk in the slide (Supplementary section TIGER AI Models). In contrast, sri and cellsvision adopted alternative solutions to use tumor segmentation to define the region to score cTILs. This suggests the importance of careful design choices on the definition of the tumor bulk region, where the concave hull approach might be too sensitive to noisy tumor segmentation outputs, and might be mitigated by improving tumor segmentation accuracy, as in the case of biototem.

### cTILs show association with response to neoadjuvant chemotherapy in HER2+ breast cancer

**Fig. 3:**
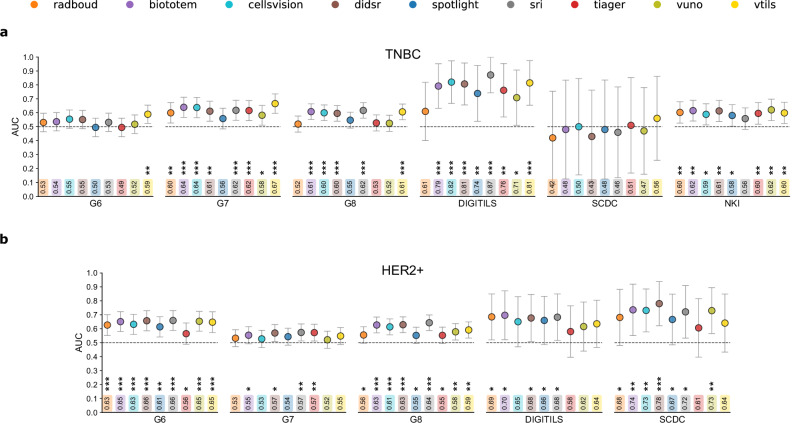
AUC for pCR prediction on biopsies. **a** Dot plots of AUC values and their 95% confidence interval for predicting pCR after NACT using cTILs and vTILs across different biopsy datasets of TNBC cases. Sample sizes: G6 (*n* = 297), G7 (*n* = 265), G8 (*n* = 390), DIGITILS (*n* = 34), NKI (*n* = 218), SCDC (*n* = 15), FinHER (*n* = 120). **b** Dot plots of AUC values and their 95% confidence intervals for predicting pCR after NACT using cTILs and vTILs across different biopsy datasets for HER2+ cases. Sample sizes: G6 (*n* = 256), G7 (*n* = 352), G8 (*n* = 368), DIGITILS (*n* = 43), SCDC (*n* = 41), FinHER (*n* = 183). Note that the NKI cohort solely contains TNBC cases. Confidence intervals and one-sided *p*-values were derived using the normal distribution approximation. Abbreviations: AUC: Area Under the Curve, NACT: neoadjuvant chemotherapy, pCR: pathological complete response, cTILs: computational tumor-infiltrating lymphocytes, vTILs: visual tumor-infiltrating lymphocytes (computed as a combination of visual TILs assessments by a group of pathologists); TNBC: triple-negative breast cancer, HER2+ : human epidermal growth factor receptor 2-positive. *p < 0.05, **p < 0.01, ***p < 0.001. Source data are provided in the source data file linked to this paper.

We benchmarked the association of cTILs with neoadjuvant chemotherapy (success of NACT), formulated as prediction of pCR treated as a binary target, and measured performance via Receiver Operating Characteristic (ROC) analysis and the Area Under the (ROC) Curve. We used cohorts of pre-operative biopsy images of patients treated with NACT from both clinical practice (NKI, DIGITILS, SCDC) and GBG clinical trials G6, G7 and G8 (Online Methods section: Materials). In TNBC (Fig. 3a), all vTILs showed AUC values > 0.5 (range [0.59–0.81]), whereas some cTILs reported AUC < 0.5 (range [0.42–0.87]). No cTILs achieved statistical significance in G6, and in SCDC neither cTILs nor vTILs showed significant association with NACT. In HER2+ (Fig. 3b), cTILs showed AUC values (range [0.52–0.78]) comparable or higher than vTILs (range [0.55–0.65]) in most cohorts. Notably, the sri and didsr methods always achieved AUC higher than vTILs in HER2+ cases and in two TNBC cohorts. Supplementary Table 7 shows the numerical data.

We ranked the algorithms by their AUC scores to compare how well each one predicted NACT response in TNBC and HER2+ cases. Results are depicted in Table 1. We observed that (a) the top-ranking AI models are those that achieve the overall higher correlation with pathologists (biototem, sri, cellsvision, didsr) (see Fig. 2c), (b) most of these models are in the top-3 positions in both TNBC and HER2+ , (c) vTILs are ranked first for TNBC but showed an overall association with NACT success lower or comparable with cTILs due to lower AUC values in HER2+ .

**Table 1:** Ranking of cTILs and vTILs based on their AUC values

TNBC		HER2+	
Methods	AUC rank (TNBC)	Methods	AUC rank (HER2+ )
vtils	1^st^	didsr	1^st^
biototem	2^nd^	biototem	2^nd^
cellsvision	2^nd^	sri	3^rd^
didsr	3^rd^	cellsvision	4^th^
sri	3^rd^	radboud	5^th^
tiager	4^th^	vtils	6^th^
vuno	5^th^	vuno	7^th^
spotlight	6^th^	spotlight	8^th^
radboud	7^th^	tiager	9^th^

*AUC* area under curve, *pCR* pathological complete response, *TNBC* triple-negative breast cancer, *HER2+* human epidermal growth factor receptor 2-positive.

Each method and visual scores of the “median” pathologist are ranked based on their AUC value for pCR prediction in the TNBC (left) and the HER2+ (right) cohorts.

### On the prognostic value of cTILs

We analyzed the prognostic value of cTILs on both pretreatment biopsies and surgical resections. From the set of 3,407 cases (see Fig. 1e) we used 3,227 cases from 7 cohorts (RUMC-TNBC, RUMC-HER2+ , FinHER, NKI, G6, G7, G8), for which survival data was available. Clinical endpoints were Disease-Free Interval (DFI) and Overall Survival (OS). All survival analyses were conducted on curated datasets comprising only patients with complete clinical and cTIL data; no missing values were imputed.

### Resections

For surgical resections, we defined two datasets: (1) RES_DFI_, containing *n* = 907 cases from RUMC-SURV_TNBC_ and FinHER, for which DFI information was available; (2) RES_OS_, containing *n* = 1128 cases from RUMC-SURV_TNBC_, RUMC-SURV_HER2+_ and FinHER, for which OS information was available.

**Fig. 4:**
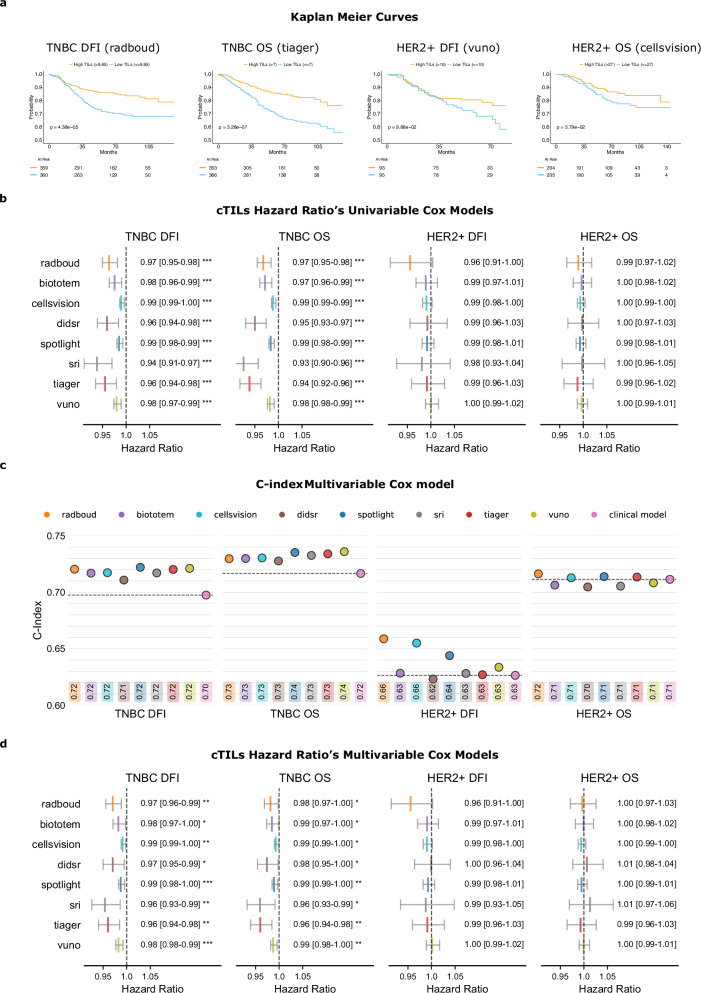
Prognostic value of cTILs as continuous values in resection samples. **a** Kaplan–Meier curves for cTIL models and survival outcomes in specific breast cancer subtypes: Radboud with DFI in TNBC (*n* = 719); Tiager with OS in TNBC (*n* = 719); VUNO with DFI in HER2+ (*n* = 188); and CellsVision with OS in HER2+ (*n* = 409). Each algorithm achieved the lowest *p*-value in its respective analysis. High and low sTILs are determined by the median cutoff value. The *P*-value was calculated by a two-sided log-rank test. **b** Forest plots of univariable Cox regression models indicating hazard ratio and 95% Confidence intervals and two-sided p-values were computed using the Wald method and depicted as HR [CI] using DFI and OS as endpoints. Sample sizes: TNBC DFI (*n* = 719), TNBC OS (*n* = 719), HER2+ DFI (*n* = 188), HER2+ OS (*n* = 409). **c** Dot plots of C-indexes computed using the prediction of a multivariable Cox regression model using a clinical model and a cTIL score as input. Values are compared with the C-index of the clinical model as a predictor (see dashed line). Sample sizes: TNBC DFI (*n* = 719), TNBC OS (*n* = 719), HER2+ DFI (*n* = 188), HER2+ OS (*n* = 409). **d** Forest plots of multivariable Cox regression models indicating hazard ratio and 95% Confidence intervals and two-sided *p*-values were computed using the Wald method and depicted as HR [CI]. Sample sizes: TNBC DFI (*n* = 719), TNBC OS (*n* = 719), HER2+ DFI (*n* = 188), HER2+ OS (*n* = 409). Plots show association for some AI models with DFI and OS on TNBC. Abbreviations: cTILs: computational tumor-infiltrating lymphocytes, TNBC: triple-negative breast cancer, DFI: disease-free interval, HER2+ : human epidermal growth factor receptor 2-positive, OS: Overall Survival. *p < 0.05, **p < 0.01, ***p < 0.001. Source data are provided in the source data file linked to this paper.

All cTILs identified two subgroups of BC patients with different prognosis via dichotomization based on per-algorithm median cTILs value, with more favorable prognosis correlating with higher cTILs. This is visible in the Kaplan-Meier (KM) curves of cTILs (Fig. 4a), only showing the results for cTILs achieving the lowest *p*-value via log-rank test on RES_DFI_ and RES_OS_, where significant difference was found in TNBC but not in HER2+ subtypes. The KM curves for all models are depicted in Supplementary section Resections - Kaplan-Meier curves. In univariable Cox regression analysis for TNBC, all cTILs showed significant correlation (*p* < 0.001) with patient outcome in both RES_DFI_ and RES_OS_. In contrast, for HER2+ , none of the models showed significant associations with either DFI or OS (Fig. 4b).

In multivariable Cox regression analyses, we first built a “clinical” model using commonly used clinicopathological variables as covariates: age, histopathological subtype, histopathological grade, molecular subtype, stage, surgical procedure and administration of adjuvant therapy. Second, we built a multivariable “enriched” model by using the output of the clinical model used as a predictor and the cTILs as a second variable (Method section Statistical analysis).

For TNBC, all multivariable-enriched models achieved a C-index (Fig. 4c) higher than the clinical model in both RES_DFI_ (C-index range across enriched models: 0.71–0.72; clinical model: 0.70) and RES_OS_ (C-index range across enriched models: 0.73–0.74; clinical model: 0.72). This shows the added value of cTILs in addition to commonly used clinical variables, also seen in the adjusted hazard ratios for the multivariable Cox analysis of continuous cTILs scores (Fig. 4d).

For HER2+ , only some enriched models achieved C-index higher than the clinical model in RES_DFI_ (C-index clinical model=0.63, C-index enriched model range [0.62–0.66]) and RES_OS_ (C-index clinical model=0.71, C-index enriched model range [0.71–0.72]) but none of them significantly added value on top of clinical variables, as shown by the adjusted hazard ratios in Fig. 4d.

### Biopsies

For preoperative biopsies, we included 2099 patients from the G6, G7, G8 and NKI cohorts (Method section Materials; Fig. 1e) for which DFI, OS and vTILs information was available, and analyzed each cohort independently. We performed survival analyses considering both cTILs and vTILs as continuous values (See Supplementary Fig.*Biopsies - Kaplan Meier Curves*, for an overview of survival curves).

**Fig. 5:**
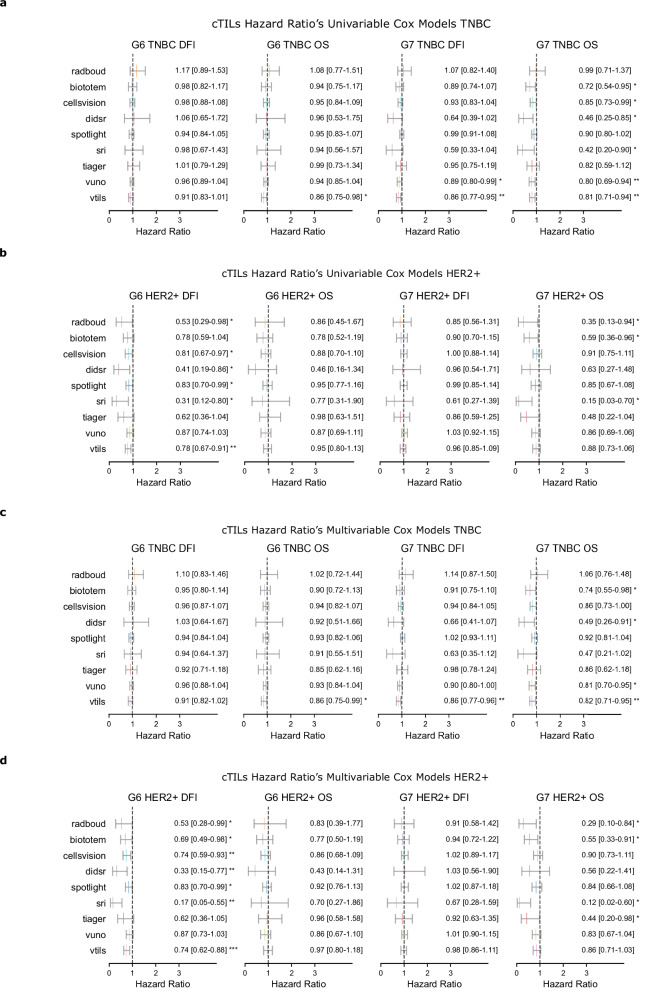
Prognostic value of cTILs in biopsy samples. **a** Forest plot for Univariable Cox regression models for all cTILs and vTILs scores for TNBC biopsies (G6 *n* = 297; G7 *n* = 260) for endpoints DFI and OS. **b** Forest plot for Univariable Cox regression models for all cTILs and vTILs scores for HER2+ biopsies (G6 *n* = 256; G7 *n* = 345) for endpoints DFI and OS. **c** Forest plot for Multivariable Cox regression models for all cTILs and vTILs scores for TNBC biopsies (G6 *n* = 289; G7 *n* = 256) for endpoints DFI and OS. **d** Forest plot for Multivariable Cox regression models for all cTILs and vTILs scores for HER2+ biopsies (G6 *n* = 251; G7 *n* = 333) for endpoints DFI and OS. Forest plots depict hazard ratios and 95% Confidence intervals and two-sided *p*-values were computed using the Wald method and depicted as HR [CI] Abbreviations: cTILs: computational tumor-infiltrating lymphocytes, TNBC: triple-negative breast cancer, DFI: disease-free interval, HER2+ : human epidermal growth factor receptor 2-positive, OS: overall survival. *p < 0.05, **p < 0.01, ***p < 0.001. Source data are provided in the source data file linked to this paper.

Overall, we found that cTILs computed on biopsies did not show a strong consistent prognostic value across cohorts and endpoints. In univariable analysis of the TNBC subset within the G7 dataset, several cTIL models demonstrated significant associations with OS (vuno, sri, didsr, cellsvision, and biototem). Furthermore, vTILs were significantly associated with OS in both G6 and G7. For DFI, only vuno and vTILS were significantly associated in G7. Figure 5a shows an overview of all Hazard Ratios for the TNBC subset of G6 and G7 datasets. In the HER2+ subset, significant associations with OS were observed in the G7 dataset for the cTILS models radboud, biototem, and sri. Regarding DFI, significant associations were identified in the G6 dataset for radboud, cellsvision, didsr, spotlight, and sri. Additionally, vTILs showed significant associations with DFI exclusively in the G6 dataset. Figure 5b shows an overview of all Hazard Ratios for the HER2+ subset of G6 and G7 datasets. No significance was observed in the G8 and NKI datasets, nor for the TNBC and HER2+ subgroups in terms of OS or DFI for any cTILs algorithm or vTILs.

Multivariable Cox analyses confirmed the significance of cTILs and vTILs observed in univariable analyses across cohorts and endpoints, except for cellsvision and sri, which were non-significant in G7 TNBC OS, vuno was not significant in G7 TNBC DFI, while tiager was significant in G7 HER2 OS, and biototem was significant in G6 HER2 DFI. Figure 5c, d show overviews of all cTILs Hazard Ratios for the TNBC and HER2+ subsets of the G6 and G7 datasets. See Supplementary Section: Biopsies - Cox Proportional Hazards Analysis for the uni- and multivariable analysis on the G8 and NKI datasets.

## Discussion

The TIGER challenge was designed with a reproducibility-first mindset, generating high-quality public sources for development and validation of AI models for cTILs quantification. These include (a) multi-centric digital pathology data to train AI models (see *Data Availability* section), (b) a public platform for benchmarking computer vision and prognostic performance of cTILs, (c) open-source baseline models, including training and testing pipelines, (d) and final AI models for cTILs quantification, available as stand-alone applications on the Grand Challenge platform and as open-source solutions with a permissive license (see Code Availability section).

The outputs of the TIGER challenge are the foundation of this study. Using the privacy-preserving Grand Challenge platform, we benchmarked TIGER cTIL models through external validation on multicentric data from routine clinical practice and phase 3 trials. We showed the strong Spearman rank correlation coefficient of most cTILs and vTILs (mean ρ value = 0.61, range [0.26–0.85]), the positive association of both cTILs and vTILs with response to neoadjuvant chemotherapy in HER2+ breast cancer (AUC cTILs range [0.52–0.78], vTILs range [0.55–0.65]), and the prognostic association of cTILs with both DFI and OS in TNBC surgical resections (multivariable C-Index range DFI [0.71,0.72], OS [0.73–0.74]). These results indicate consistent improvements across independently developed cTIL models above already strong performance of the baseline clinical model. If such cTIL models were to be adopted in clinical settings, even small improvements in predictive accuracy could help further stratify patients and potentially influence treatment decisions or follow-up strategies. Besides these results, we also showed the poor association between cTILs and survival (DFI and OS) on HER2+ surgical resections and on preoperative biopsies. The cohorts used in TIGER as training and test data contained higher numbers of TNBC compared to HER2+ cases, as well as more surgical resection slides than biopsies. Although this information was not disclosed to the TIGER participants, these factors may have implicitly steered models’ optimization towards performance on TNBC resections during the experimental phase of the challenge. While the inclusion of data from multiple institutions and countries contributed to mitigate certain biases and improve model robustness, differences in patient populations, and institutional cohorts are factors known to influence model performance. Access to larger and more diverse training datasets, particularly with more preoperative biopsies, will be part of future work to balance optimization and reduce potential bias that remains across clinical settings.

While an accurate analysis of tissue morphology remains the base for robust cTILs scores, we found a discrepancy between methods achieving high computer vision performance (tiager, biototem, spotlight), cTILs showing strong correlation with pathologists (biototem, sri, cellsvision), and association with pCR and survival. We found that methods that automated the recommendations of the TILs WG^2^ reported Spearman rank correlation coefficient with vTILs ranging from weak (ρ = 0.26) to very strong (ρ = 0.85), especially for biopsies. This may indicate the importance of algorithmically defining the region where TILs are scored, since methods strongly relying on the definition of the tumor bulk (radboud, tiger, spotlight) or on peritumoral regions (vuno) showed lower correlation with visual TILs and lower association of cTILs with pCR computed on biopsies. This suggests that the optimal way to engineer spatial morphology in cTILs models may differ from visual scoring by pathologists and remains an open research question. Beyond algorithmic factors, biological heterogeneity may also contribute to performance differences. Future research should investigate whether tumor microenvironment heterogeneity and molecular subtype characteristics, e.g., ER status stratification within HER2+ cases, contribute to differential cTILs performance.

Our study provides insights for both researchers and clinicians. For researchers, we provide indications on how to develop cTILs methods, addressing the lack of consensus methods on cTILs design, fostering further development and validation of improved AI models for cTILs in the future. For clinicians, we introduced a way to obtain indirect access to models via Grand Challenge to test automated cTILs assessment on anonymous data for research use. Our results also suggest that cTILs could be a useful tool to assist pathologists in TILs quantification. Compared to visual TILs assessment, cTILs are more objective and reproducible, can provide visual feedback on tissue segmentation and lymphocyte detection and support decision making of pathologists with interpretable visual output. These potential advantages may be particularly relevant in settings with limited access to specialized pathology expertise .

**Table 2:** Table-overview-post-challenge-cohorts

Cohort	Tissue	Scanner	Magnification	micron/px	Country	Source
RUMC-SURV	resections	Pannoramic 1000 DX	40X	0.242	Netherlands	clinic
FinHER	resections	NanoZoomer 2.0-RS C10730	40X	0.228	Finland	trial
GBG (G6, G7, G8)	biopsies	Leica Aperio AT2 Sysmex NanoZoomer 2.0-HAT	20X	0.503	Germany	trial
GBG (G6, G7, G8)	biopsies	Pannoramic 250 FLASH III Pannoramic SCAN II	40X	0.242	Germany	trial
NKI	biopsies	Aperio AT2	40X	0.252	Netherlands	trial
DIGITILS	biopsies	Nanozoomer 2.0RS	40X	0.227	Belgium	clinic
SCDC	biopsies	Ventana DP 200	20X	0.465	Italy	clinic

Overview of cohorts used for the post-challenge validation, including details on data type, scanner, magnification, spacing (in micron/px), country and whether the data comes from daily clinical practice or from clinical trials. Magnification indicates the one used originally to scan the slides, all WSI were then processed at 0.5 μm/px in this work.

*RUMC* Radboud University Medical Center, *GBG* German Breast Group, *NKI* Nederland Kanker Instituut (Dutch Cancer Institute), *SCDC* Sacro Cuore Don Calabria hospital.

Future work should explore how cTILs can be integrated into clinical workflows and assess their impact on pathologist decision-making. This could include prospective studies evaluating clinical utility, human-AI interaction, and workflow efficiency, as well as implementation research within routine digital pathology systems used in diagnostic practice. The path towards clinical adoption of these solutions will require further validation on both retrospective and prospective data. In this context, the privacy-preserving mechanism for indirect access to data (i.e., without sharing data with AI developers) and the public benchmark built within TIGER have the potential to become an easily-accessible benchmark for validation of present and future cTILs methods, with potential value to support certification for regulatory purposes.

Compared to TIGER, in this study we have broadened the statistical analysis across multiple cohorts and clinical endpoints (pCR, DFI, OS). Accordingly, we have expanded the TIGER web platform to include the additional statistical tests used in this study. Furthermore, international reader studies, in addition to those already performed^6,35^, could increase our evidence on inter-observer variability and possibly corroborate the need for computational quantification of the TILs. Alongside, research on the usability and impact of cTIL models are needed to better understand their potential as a computer-aided supportive tool and to confirm whether AI models maintain robust performance across the full spectrum of breast cancer encountered in routine diagnostics. Incorporating a pathologist-in-the-loop workflow, where low model confidence or out-of-distribution cases are flagged for expert review, may provide an additional safeguard and facilitate model integration into the clinic.

In addition to informing future clinical studies, our benchmarking framework and the open sharing of data, code, and evaluation procedures directly support key regulatory requirements by enabling transparent, reproducible comparisons of cTIL models across diverse datasets and providing a basis for potential approval for clinical use.

### Limitations

This study has limitations. First, our validation was based on a mechanism of indirect access to data, which did not allow us to investigate the causes of differences in computer vision performance and predicted cTILs. Examples are segmentation outputs on specific regions of WSIs that play a role in the tumor microenvironment, such as tertiary lymphoid structures, lymphoid stroma, perivascular invasion, and other confounders known to play a role in visual TIL scoring, which could not be fully explored. These aspects deserve a more detailed analysis and should be addressed in dedicated future studies.

Second, we analyzed raw cTILs values as produced by AI models, without performing any form of calibration. In the roadmap towards assessing the clinical applicability of computational biomarkers and their potential complementarity with visual scores, research on calibration of cTILs scores in line with what recently proposed by Arab et al.^36^ will be needed.

Third, the TIGER challenge instructed participants to produce models producing cTILs scores within a range between 0 and 100. However, each cTILs algorithm produced scores with a different distribution. As a consequence, it was not possible to dichotomize groups of patients using cut-off values such as 30%^7^ or 10–60%^9^, used in previous studies. Therefore, in Kaplan-Meier analyses, we considered the median cTILs value per cohort as a cut-off point to dichotomize subgroups of patients. Next to calibration, future work will require the estimation of per-algorithm optimal cut-off points.

Fourth, this study did not identify a clear winner among the evaluated methods across all considered end points. Instead, we identified the potential of several AI assays to target particular clinical endpoints and breast cancer subtypes based on the data at hand. Despite efforts to address variability in scanning and staining by including cases from multiple centers and technologies, heterogeneity in image quality, staining intensity, and scanner characteristics exceeds any curated dataset.

Finally, we limited the analysis to the association of cTILs with response to (neoadjuvant) chemotherapy. Given the increasing use of immune checkpoint inhibitors in combination with chemotherapy in early-stage TNBC, future development and validation of AI-based TILs should extend to treatment regimens including immune-checkpoint inhibitors. Moreover, given the previously demonstrated excellent long-term clinical outcomes of patients with anatomical low-stage TNBC and high TILs, even in the absence of chemotherapy, the potential for AI-based TIL measures to select patients for de-intensification is a compelling application that requires dedicated prospective evaluation.

In conclusion, we have presented the analysis of several cTIL models on a multi-centric cohort of digital pathology whole-slide images of breast cancer patients. We have reported benchmarks on image analysis performance of each method, showed the agreement of cTILs with panels of pathologists, the positive association of cTILs with response after neoadjuvant therapy, and revealed a trend of achieving higher AUC values than pathologists in HER2+ cases. We showed that cTILs are associated with survival both as a stand-alone biomarker and by adding independent information to clinical variables in multivariable analyses in surgically resected TNBC, but not in HER2-positive disease. Finally, we showed that cTILs on biopsies did not show a strong consistent prognostic value across cohorts and endpoints. All methods tested in this work originated in an open community-driven project based on the TIGER challenge. This work demonstrates public development and validation of cTIL models using artificial intelligence. This collaborative and transparent approach may help accelerate the development of computational biomarkers more broadly, and serves as a step towards reproducible and community-driven biomarker research.

## Methods

### Inclusion and ethics

Patients gave written informed consent, opted-in, or did not opt-out (according to local clinical regulations) for the use of tissue for research purposes, which was approved by the ethical committees for all clinical cohorts and clinical trials. The Committee on Research Involving Human Subjects of the Radboud University Medical Center (Radboudumc) has approved the use of data from the RUMC-TNBC and RUMC-HER2+ cohorts (case number 2015–1711). For the FinHer trial, an ethics committee at Helsinki University Hospital approved the study (trial identifier: ISRCTN76560285). For DIGITILS, the institutional ethics committee approved this study (file number: RETRO-TNBC-15-2019/03JUL/297 for the TNBC cohort, file name: RETRO-HER2-15 for the HER2+ cohort). The approval to use slides from the GBG was given at two institutions: Charité ethic committee approval number: EA1/139/05; Philipps-University Marburg ethic committee approval number: 38/20. The use of the slides from SCDC for the study was approved by the Ethics Committee for Clinical Research of the Provinces of Verona and Rovigo under number 25046. The use of the slides from NKI for the study was approved by the institutional review board of the Netherlands Cancer Institute under number CFMPB737.

### Materials

In this section, we introduce all cohorts involved in cTILs benchmarking in this study and report the type of annotations available for each cohort. We refer to the Supplementary material for TIGER training data and computer vision benchmark data, including the re-staining study. All slides were stained with H&E, scanned in the origin center with whole-slide image scanners locally available and processed to standard TIFF at 20X magnification (0.5 μm/px).

### TIGER models

All submitted methods were based on Convolutional Neural Network (CNN) architectures. Most teams used standard segmentation backbones (e.g., U-Net, UNet + +, or DeepLab) for tumor and stroma segmentation, and object detection components (e.g., YOLO or Faster R-CNN, typically with ResNet or DenseNet encoders) for lymphocyte detection. Each algorithm produced a cTIL score on a 0–100 scale by aggregating lymphocytes within a region, defined either by explicit tumor or stroma segmentation, or by a tumor-bulk outline. Per-algorithm details are provided in Supplementary Materials: TIGER Models.

### Definition of clinical outcome

#### Pathological complete response

In all cohorts, pCR was based on the local histopathological analysis of the resection specimen after neoadjuvant chemotherapy and indicated the absence of invasive cancer in the breast and axillary nodes, which is our primary target. GBG and SCDC defined pCR as ypT0/ypN0 (i.e., absence of in-situ cancer) but using different scoring systems (see Data for details); NKI and DIGITILS defined pCR as ypT0/is ypN0 (irrespective of ductal carcinoma in-situ as this information was not available).

#### Survival

We used the following definitions of survival data: disease-free interval (DFI), defined as the time in months between diagnosis (for clinical cases) or randomization (for clinical trials) and the date of clinically and/or pathologically detected (loco)regional or distant recurrence of BC. If no recurrence occurred, patients were censored at the date of last follow-up, death, or secondary malignancy. Overall survival (OS) is defined as the time in months between date of diagnosis of BC and date of death (independent of cause) or the date of last follow up (right-censored).

### Data

#### RUMC-SURV_TNBC_

This cohort consisted of *n* = 597 TNBC cases (stage 1–3), derived from a previous study^37^. In brief, TNBC cases diagnosed between 2006 and 2014 in five hospitals from Eastern Netherlands (Radboudumc, Nijmegen; Canisius-Wilhelmina Hospital, Nijmegen; Jeroen Bosch Hospital, ‘s-Hertogenbosch; Bernhoven Hospital, Uden and Hospital Pantein, Boxmeer) were included. At the time of inclusion, patients had not received neoadjuvant chemotherapy and did not have prior history of breast cancer. All tissue blocks were collected centrally and cut and stained in batches in the pathology laboratory of the Radboudumc. For this reason, although patient data is multi-centric, we refer to this cohort as the RUMC-SURV_TNBC_ cohort. All slides were scanned with a Pannoramic 1000 DX scanner (3DHistech) at 40X magnification (0.24 μm/px). For each patient, the following clinicopathological information was available: age, histological subtype, grade, molecular subtype, stage, surgery, adjuvant therapy. For this cohort, DFI and OS end-points were available, visual assessment of stromal TILs (sTILs) was not available.

#### RUMC-SURV_HER2+_

This cohort consisted of *n* = 221 HER2+ cases (stage 1–3) collected between 2006 and 2013 at two Dutch hospitals (Radboudumc, Nijmegen; Canisius-Wilhelmina Hospital, Nijmegen). As for RUMC-SURV_TNBC_, all tissue blocks were collected, cut, stained and scanned at Radboudumc; therefore, we refer to this cohort as the RUMC-SURV_HER2+_. Patients did not get any neoadjuvant treatment, did not have multiple breast tumors at the time of diagnosis nor bilateral breast cancer, did not have multiple diagnosis of breast or other invasive tumors within 5 years, and all of them received treatment. HER2+ status was based on immunohistochemistry (3+ score) and/or FISH (amplification). Scanner settings and clinicopathological information were the same as RUMC-SURV_TNBC_. For this cohort, only OS follow-up data was available, sTILs assessment was not available.

### FinHER

This cohort consisted of *n* = 303 cases from the FinHer trial^38^. Slides were stained with H&E and scanned at the Jules Bordet Institute (Belgium) using a NanoZoomer 2.0-RS C10730 series (Hamamatsu) at a resolution of 0.23   μm/px. From the original FinHer cohort, we selected TNBC (*n* = 122) and HER2+ (*n* = 188) cases, after excluding cases with incomplete data (e.g., no match between image data and clinical data) and cases with stage 4 disease at initial presentation. Both HR status and HER2 expression were determined by immunohistochemistry according to each institution’s guidelines. When HER2 expression was considered positive in immunohistochemistry (either 2 or 3 on a scale from 0 to 3), gene amplification status was determined centrally using CISH. Cancers with six or more gene copies were considered HER2 positive. The same clinicopathological information as RUMC-SURV_TNBC_ and RUMC-SURV_HER2_ were available. DFI and OS data were available as endpoints, as well as visual sTIL assessment scored by two breast pathologists (RS, NS).

### GBG

We included a set of *n* = 1928 patients with primary breast cancer who were treated with neoadjuvant combination chemotherapy from three randomized trials done by the German Breast Cancer Group (GBG), namely GeparSixto^39^ (G6), GeparSepto^40^ (G7), GeparOcto^41^ (G8). All patients underwent core-needle biopsy prior to breast cancer diagnosis and subsequent neoadjuvant chemotherapy treatment. All 1928 patients contributed to the pCR analyses. For DFI and OS, the evaluable numbers (TNBC, HER2+ ) were 297 and 256 in G6, 260 and 345 in G7, and 390 and 368 in G8 for univariable analyses (total *n* = 1916), and 289 and 251 in G6, 256 and 333 in G7, and 386 and 362 in G8 for multivariable analyses (total *n* = 1877). Differences reflect unavailable outcome data and missing clinical variables required for multivariable adjustment. Slides were stained and scanned at the Institute of Pathology, Philipps-University Marburg, University Hospital UKGM Marburg using either a Leica Aperio AT2, a Sysmex/3DHistech’s Pannoramic 250 FLASH III and Pannoramic SCAN II or a NanoZoomer 2.0-HAT from Hamamatsu. The following clinicopathological variables were available for these cohorts assessed at baseline: pathological grading, molecular subtype, calculated on the basis of HR (ER, PR) and HER2 status, Ki67 value, tumor size (T stage) and nodal status (N stage). For these cohorts, DFI and OS data were available as endpoints; pCR was defined as ypT0/ypN0, sTILs were available and scored as described in ref. ^9^.

### DIGITILS

We included a set of *n* = 77 cases from the Cliniques Universitaires Saint-Luc (Brussels, Belgium), *n* = 34 TNBC and *n* = 43 HER2+ . All patients underwent core-needle biopsy prior to breast cancer diagnosis and subsequent neoadjuvant chemotherapy treatment. The TNBC part of this cohort belongs to the IVITA study^6^, involving a reader study with *n* = 40 pathologists per slide that assessed sTILs. The HER2+ part belongs to a recent study^35^, involving a reader study with *n* = 3 pathologists per slide. All slides were scanned at Saint Luc Hospital using a Hamamatsu Nanozoomer 2.0RS scanner at 40X magnification. For each patient, available information contained tumor subtype, tumor grade, and response to neoadjuvant chemotherapy scored based on the Residual Cancer Burden (RCB) scoring system (RCB = 0 corresponds to pCR defined as ypT0/is ypN0).

### NKI

We included a set of *n* = 218 TNBC routine diagnostics cases from the Dutch Cancer Institute (Nederland Kanker Instituut, NKI; Amsterdam, Netherlands). All patients underwent core-needle biopsy prior to breast cancer diagnosis and subsequent neoadjuvant chemotherapy treatment. All slides were stained at the NKI and scanned with an Aperio AT2 (Leica Biosystems) at 40X magnification. The same clinicopathological variables used in GBG were available; DFI and OS data were available as end points; pCR was defined as ypT0/is ypN0, meaning absence of invasive cancer in breast and axillary nodes irrespective of ductal carcinoma in-situ as this information was not available; sTILs were scored by two pathologists following the recommendations of the TIL WG, as described in ref. ^42^.

### SCDC

We included *n* = 56 cases (15 TNBC, 41 HER2+ ) from the IRCCS Sacro Cuore Don Calabria Hospital (SCDC, Verona, Italy). All slides are diagnostic biopsies stained with H&E, extracted via core-needle procedure (before chemotherapy), and scanned with a Ventana DP 200 slide scanner at 20X magnification. The same clinical variables and clinical outcomes included in the GBG and NKI cohorts were available, with the exception of T stage. However, due to the limited number of events (*n* = 11 recurrence, *n* = 6 death) and the high rate of censored cases (*n* = 41 (73%), for no recurrence and no death), we only considered this cohort for the correlation with pathologists and the prediction of pCR. pCR was derived from the PINDER scoring system^43^ (PINDER=1i for breast and PINDER = 1 + 2 for lymph nodes). sTILs were scored by one pathologist (EM) following the recommendations of the TIL WG.

### Statistics and reproducibility

This study used retrospective cohorts defined in previous studies. Sample size had been determined by case availability in the participating cohorts and clinical trials; therefore, no statistical method was used to predetermine sample size in our study. Randomization was not applicable. The TIGER challenge used a privacy-preserving evaluation framework in which AI models were tested on sequestered datasets inaccessible to participants. Outcome assessment followed a pre-specified statistical analysis plan.

#### Computer vision performance

We benchmarked tissue segmentation and lymphocyte detection with the same approach used in the computer vision track of TIGER.

##### Segmentation

For tissue segmentation, we focused on two classes that play a central role in the definition of the TILs score and computed an overall Dice score as the average of the Dice score for tumor and the Dice score for tumor-associated stroma. For this, we created a test set with pixel-level annotations (DET_SEG_) based on ROIs delineated by breast pathologists. Within each ROI, every pixel was annotated into one of seven classes: invasive tumor, tumor-associated stroma, in-situ tumor, healthy glands, necrosis (non-in-situ), inflamed stroma, and a ‘rest’ category (a heterogeneous group comprising of fat cells, erythrocytes, artifacts, and other occurring patterns). For the computation of the DICE score, these annotations were mapped to two target classes: (i) invasive tumor and (ii) tumor-associated stroma, while all remaining categories were merged into a single non-target class. Dice scores were computed across all ROIs in the test slides by treating the pixels of the class of interest (either invasive tumor or tumor-associated stroma) as foreground and all other annotated pixels within the same ROI as background.

##### Detection

For lymphocyte detection, we performed a Free Response Operating Characteristic (FROC) analysis, computing sensitivity (true positive rate, TPR) versus average false positives (FP) per mm² over all slides. Given the i-th predicted cell locations ${c}_{p,i}=({x}_{p,i},{y}_{p,i})$ of lymphocytes and plasma cells, each with likelihood *l*_*i*_, and the j-th manual annotation ${c}_{m,j}=\left({x}_{m,j},{y}_{m,j}\right)$, we considered a hit of manual annotation ${c}_{m,j}$ if$\,{l}_{i} > \tau$ and if $d\left({c}_{p,i},{c}_{m,j}\right)\le D$, where $\tau$ is a threshold and $d\left(\cdot \right)$ is the Euclidean distance. In our case, $\tau$ assumed all likelihood values predicted by each algorithm on the entire test set, and D=4um, based on previous work. For each threshold $\tau$, we obtained values for the true positives (TPs), false negatives (FNs), and false positives (FPs) and used those to build an FROC curve. From the curve, we derived a “FROC score” by averaging sensitivity computed at six pre-selected values of FP/mm²: (10, 20, 50, 100, 200, 300).

### Combination of sTILs

In several cohorts (NKI, DIGITILS, GBG, FinHER, SCDC), one or more pathologists visually assessed the stromal TILs according to recommendations of the TIL working group. The analysis of the inter-observer variability was addressed in previous studies, including the ones in which the used cohorts were originally introduced^6,35,38–41^ and is therefore out of scope for this study. Here, following^6^, we combined the multiple sTILs scores at slide level into a single score by computing the *median* sTILs score. Note that a) this will result in the mean sTILs value when only two pathologists were involved; b) we combined the sTILs score without any transformation, because following the TILs WG recommendations, each score indicates the percentage of stroma covered by the TILs in the tumor bulk, therefore producing a value between 0 and 100. We refer to this combined score as the pathologist visual TILs (vTILs). When multiple slides per patient were available, one representative slide was selected being the one with the highest vTILs score.

#### Correlation with vTILs

We measured the correlation between each cTIL score and the vTILs. We report correlation metrics in terms of Spearman rank correlation coefficient and via scatter plots for each pair of TIL scores (see Fig. 2c). Possible discretized pathologist scores and cTILs distributions not necessarily aligned with the ones of visual scoring make the Spearman rank correlation coefficient appropriate for capturing monotonic relationships between cTIL and vTIL scores.

#### Treatment response: prediction of pCR

Based on previous studies, we assume that high TILs predict pCR while low TILs predict no pCR. For cohorts of pre-treatment biopsies of patients that received neoadjuvant chemotherapy (NKI, GBG, DIGITILS, SCDC), we performed Receiver Operating Characteristic (ROC) curve analysis for all cTILs and vTILs using pCR as a target. From each ROC curve, we computed the area under the curve (AUC) as the performance metric as well as its confidence interval. We also computed a one-sided Wald *p*-value from a normal distribution approximation. No adjustments were made for testing multiple hypotheses.

#### Prediction of survival

Based on previous studies, we assumed that high TILs predict longer survival and low TILs predict a higher risk of an event. We tested this hypothesis using cTILs from all algorithms and vTILs on cohorts with survival data available (NKI, GBG, FinHER, RUMC-SURV_TNBC_, RUMC-SURV_HER2+_). We detail here the tests that we performed on cohorts containing surgical resections and biopsies. Partly derived from the survival task of TIGER, we redesigned the survival analysis performed in this study to consider additional tests and endpoints. Adjustments for multiple hypothesis testing were not performed, as each AI-based cTIL method was independently assessed for its prognostic significance and comparisons were made based on their relative effect sizes and associated *p*-values descriptively.

### Prediction based on surgical resections

For surgical resections, we tested the prognostic value of cTILs in addition to the clinical variables that we considered in the TIGER challenge, namely molecular subtype, age, morphological subtype, grade, stage, adjuvant treatment. We did it in three steps. First, we pooled together all cases from FinHER, RUMC-SURV_TNBC_ and RUMC-SURV_HER2+_, resulting in a cohort of *n* = 1128 cases, which we called ALL-SURV. Second, we built a multivariate Cox regression by fitting the model on all clinical covariates of ALL-SURV and we saved this model and turned it into a predictor, which we refer to as the clinical model, to use it in downstream tasks. Third, for each algorithm, we updated the model by replacing all the previously described clinical covariates with the predictor, which was common to all algorithms, and enriched it with cTILs as the second covariate in the updated multivariable Cox regression model; we refer to this model as the enriched model. By using the same clinical predictor in all models, we ensured fair comparisons of the added prognostic value of each cTILs algorithm. We also used the output of the enriched model to compute the C-index using either DFI or OS as end points. For this, we used the method proposed by Harrel^44^, commonly used in the literature. Note that this is different from the C-index implementation adopted during the TIGER challenge, which was based on the Uno’s AUC approach (Supplementary section Survival track evaluation). Univariable analysis of cTILs is done via Kaplan-Meier curves analysis, and *p*-values computed via the log-rank test. Hazard ratios depicted in the forest plots were obtained from univariable Cox models. In our analysis, we considered TILs scores as a continuous value and *p*-values for the hazard ratios were derived from the Wald test.

### Prediction based on pretreatment biopsies

For biopsies, we assessed the prognostic value of cTILs on OS and DFI in a univariable setting (Kaplan Meier curves and Cox regression model) and multivariable setting (Cox regression model). In Kaplan Meier curves, for each algorithm, categories high and low were dichotomized based on the dataset-specific median value analyzed in the respective section, namely G6, G7, G8 or NKI. For each endpoint and for each algorithm (and vTIL), we calculated the cTIL hazard ratio (or vTIL, respectively) from a univariable and multivariable (with covariates: molecular subtype, grade, stage (T, N), TIL score) Cox regression model that was run over each biopsy cohort in which survival data were available. Corresponding cTILs were handled as a continuous variable in the Cox regression models. *P*-values for the hazard ratios were derived from the Wald test.

### Reporting summary

Further information on research design is available in the Nature Portfolio Reporting Summary linked to this article.

## Supplementary information

Supplementary Information

Reporting Summary

Transparent Peer Review file

## Source data

Source Data

## Data Availability

The minimum dataset required to interpret and reproduce the analyses in this study consists of histopathology whole-slide images and corresponding annotations used in the TIGER challenge and external validation sets. Public training data are available via the AWS Open Data Registry (https://registry.opendata.aws/tiger/), which hosts the full set of training whole-slide images and manual annotations for the WSIROI, WSIBULK and WSITILS subsets. A size-reduced subset of the training data, containing regions of interest from the WSIROI subset and corresponding annotations, is archived in Zenodo under DOI: 10.5281/zenodo.6014422. The TIGER-CV and TIGER-SURV test datasets are not directly accessible to preserve the integrity of the benchmark and are available via indirect (i.e., restricted) access through model evaluation by submitting algorithms via the Grand Challenge platform (https://tiger.grand-challenge.org). Access is limited to model evaluation; raw data are not downloadable. Joining the platform using a verified user account and submitting a model gives immediate indirect access to the data. Data are available for the duration of the platform’s operation for at least five years since the launch of the challenge. Data from the GBG, NKI, DIGITILS and SCDC cohorts consist of histopathology images and associated clinical outcome data are subject to ethical approval, patient consent, and institutional data-use agreements. Access to these datasets can be requested directly through the respective data-owning clinical trial groups or institutions. Requests for GBG should be directed to Trafo@gbg.de. Requests for NKI should be directed to Esther Lips (e.lips@nki.nl). Requests for DIGITILS should be directed to Mieke van Bockstal (mieke.vanbockstal@saintluc.uclouvain.be). Requests for SCDC should be directed to Giuseppe Bogina (giuseppe.bogina@sacrocuore.it). Source data are provided with this paper.

## Code availability

Information about code used in the TIGER challenge is available on the TIGER website (https://tiger.grand-challenge.org/Code/). The code of the baseline TIGER algorithm developed by the Computational Pathology Group of Radboudumc is available at https://github.com/DIAGNijmegen/pathology-tiger-baseline. An example of making a docker container to upload an algorithm to the TIGER platform on Grand Challenge is available at https://github.com/DIAGNijmegen/pathology-tiger-algorithm-example. TIGER evaluation metrics are available at https://github.com/DIAGNijmegen/pathology-tiger-algorithm-example/tree/main/evaluations, including code to benchmark computer vision and survival performance. The WholeSlideData package(v0.0.16) used to read and write WSIs data within TIGER are available at https://github.com/DIAGNijmegen/pathology-whole-slide-data. The source code and binaries of the ASAP viewer are available at https://github.com/computationalpathologygroup/ASAP (v2.1). The Grand Challenge platform is an open-source code, available at https://github.com/comic/grand-challenge.org/. All TIGER models are publicly available under permissive licenses and are available at: aivis (https://github.com/AIVIS-MING/TIGER_SEG-DET). biototem (https://github.com/biototem/TIGER_challenge_2022). cellsvision (https://github.com/XulinChen/Algorithm-for-Tiger-Challenge). didsr (https://github.com/DIDSR/DIDSR-TiGER). radboud (https://github.com/DIAGNijmegen/pathology-tiger-baseline). spotlight (https://github.com/Spotlight-Pathology/spotlight-tiger). sri (https://github.com/Vishwesh4/TigerSubmission). tiager (https://github.com/adamshephard/TIAger). vuno (https://github.com/vuno/tiger_challenge).
