# Supplementary material for: Analysis of computational tumor-infiltrating lymphocytes in breast cancer from the results of the TIGER challenge
Source: Nat Commun. 2026 May 15;17:6480. doi: 10.1038/s41467-026-72956-x (PMC13377072; doi:10.1038/s41467-026-72956-x)
Supplement: Supplementary file 2 — Reporting Summary [file 41467_2026_72956_MOESM2_ESM.pdf]

nature portfolio

Corresponding author(s): Francesco Ciompi
Last updated by author(s): 20-02-2026, Francesco Ciompi, Mart van Rijthc

Reporting Summary

Nature Portfolio wishes to improve the reproducibility of the work that we publish. This form provides structure for consistency and transparency in reporting. For further information on Nature Portfolio policies, see our Editorial Policies and the Editorial Policy Checklist.

Statistics

For all statistical analyses, confirm that the following items are present in the figure legend, table legend, main text, or Methods section.

n/a	Confirmed	
☐	☒	The exact sample size (*n*) for each experimental group/condition, given as a discrete number and unit of measurement
☐	☒	A statement on whether measurements were taken from distinct samples or whether the same sample was measured repeatedly
☐	☒	The statistical test(s) used AND whether they are one- or two-sided<br>*Only common tests should be described solely by name; describe more complex techniques in the Methods section.*
☐	☒	A description of all covariates tested
☐	☒	A description of any assumptions or corrections, such as tests of normality and adjustment for multiple comparisons
☐	☒	A full description of the statistical parameters including central tendency (e.g. means) or other basic estimates (e.g. regression coefficient) AND variation (e.g. standard deviation) or associated estimates of uncertainty (e.g. confidence intervals)
☐	☒	For null hypothesis testing, the test statistic (e.g. *F*, *t*, *r*) with confidence intervals, effect sizes, degrees of freedom and *P* value noted<br>*Give P values as exact values whenever suitable.*
☒	☐	For Bayesian analysis, information on the choice of priors and Markov chain Monte Carlo settings
☐	☒	For hierarchical and complex designs, identification of the appropriate level for tests and full reporting of outcomes
☐	☒	Estimates of effect sizes (e.g. Cohen's *d*, Pearson's *r*), indicating how they were calculated

Our web collection on statistics for biologists contains articles on many of the points above.

Software and code

Policy information about availability of computer code

Data collection	Manual training annotations were created by multiple annotator groups. For TCGA-BRCA slides, tissue annotations and cell annotations originated from the BCSS and NuCLS projects, for which a crowdsourcing platform was created to annotate tissue compartments and cell locations. For RUMC-CV and Jules Bordet slides, tissue–cell annotations were produced using the CIRRUS Pathology web viewer and reader-study functionalities available on the Grand Challenge platform. Training annotations were released in XML/TIFF formats and can be visualized by loading the XML files in the ASAP viewer (v2.1). For the re-staining subset (DETRESTAIN), H&E and IHC whole-slide images were registered using HistokatFusion, and lymphocyte annotations were made on IHC slides and mapped to H&E using the registration.
Data analysis	Benchmarking and analyses were performed through the TIGER/Grand Challenge infrastructure by executing dockerized algorithm submissions on sequestered datasets and computing evaluation outputs. Information about code used in TIGER is available on the TIGER website (https://tiger.grand-challenge.org/Code/). The TIGER baseline algorithm is available at https://github.com/DIAGNijmegen/pathology-tiger-baseline. An example docker container for uploading an algorithm is available at https://github.com/DIAGNijmegen/pathology-tiger-algorithm-example. TIGER evaluation metrics (computer vision and survival benchmarking) are available at https://github.com/DIAGNijmegen/pathology-tiger-algorithm-example/tree/main/evaluations. The WholeSlideData packages used to read and write WSIs within TIGER are available at https://github.com/DIAGNijmegen/pathology-whole-slide-data (v0.0.16). The source code and binaries of the ASAP viewer are available at https://github.com/computationalpathologygroup/ASAP. The Grand Challenge platform is open-source at https://github.com/comic/grand-challenge.org/. All TIGER models are publicly available under permissive licenses:<br>aivis (https://github.com/AIVIS-MING/TIGER_SEG-DET),<br>biototem (https://github.com/biototem/TIGER_challenge_2022),<br>cellsvision (https://github.com/XulinChen/Algorithm-for-Tiger-Challenge),<br>didsr (https://github.com/DIDSR/DIDSR-TiGER),<br>radboud (https://github.com/DIAGNijmegen/pathology-tiger-baseline),

spotlight (https://github.com/Spotlight-Pathology/spotlight-tiger),
sri (https://github.com/Vishwesh4/TigerSubmission),
tiger (https://github.com/adamshephard/TIAger),
vuno (https://github.com/vuno/tiger_challenge).

For manuscripts utilizing custom algorithms or software that are central to the research but not yet described in published literature, software must be made available to editors and reviewers. We strongly encourage code deposition in a community repository (e.g. GitHub). See the Nature Portfolio guidelines for submitting code & software for further information.

Data

Policy information about availability of data

All manuscripts must include a data availability statement. This statement should provide the following information, where applicable:

- Accession codes, unique identifiers, or web links for publicly available datasets
- A description of any restrictions on data availability
- For clinical datasets or third party data, please ensure that the statement adheres to our policy

The minimum dataset required to interpret and reproduce the analyses in this study consists of histopathology whole-slide images and corresponding annotations used in the TIGER challenge and external validation sets. Public training data are available via the AWS Open Data Registry (https://registry.opendata.aws/tiger/), which hosts the full set of training whole-slide images and manual annotations for the WSIROI, WSIBULK and WSITILS subsets. A size-reduced subset of the training data, containing regions of interest from the WSIROI subset and corresponding annotations, is archived in Zenodo under DOI: 10.5281/zenodo.6014422. The TIGER-CV and TIGER-SURV test datasets are not directly accessible to preserve the integrity of the benchmark and are available via indirect access through model evaluation by submitting algorithms via the Grand Challenge platform (https://tiger.grand-challenge.org). Access is limited to model evaluation; raw data are not downloadable. Joining the platform and submitting a model gives immediate indirect access to the data for the purpose of model evaluation. Data are available for the duration of the platform's operation for at least five years since the launch of the challenge. Data from the GBG, NKI, DIGITILS and SCDC cohorts consist of histopathology images and associated clinical outcome data are subject to ethical approval, patient consent, and institutional data-use agreements. These datasets are available under restricted access and can be requested directly through the respective data-owning clinical trial groups or institutions. Requests for GBG should be directed to Trafo@gbg.de. Requests for NKI should be directed to Esther Lips (e.lips@nki.nl). Requests for DIGITILS should be directed to Mieke van Bockstal (mieke.vanbockstal@saintluc.uclouvain.be). Requests for SCDC should be directed to Giuseppe Bogina (giuseppe.bogina@sacrocuore.it).

Research involving human participants, their data, or biological material

Policy information about studies with human participants or human data. See also policy information about sex, gender (identity/presentation), and sexual orientation and race, ethnicity and racism.

Reporting on sex and gender	*Use the terms sex (biological attribute) and gender (shaped by social and cultural circumstances) carefully in order to avoid confusing both terms. Indicate if findings apply to only one sex or gender; describe whether sex and gender were considered in study design; whether sex and/or gender was determined based on self-reporting or assigned and methods used. Provide in the source data disaggregated sex and gender data, where this information has been collected, and if consent has been obtained for sharing of individual-level data; provide overall numbers in this Reporting Summary. Please state if this information has not been collected. Report sex- and gender-based analyses where performed, justify reasons for lack of sex- and gender-based analysis.*
Reporting on race, ethnicity, or other socially relevant groupings	*Please specify the socially constructed or socially relevant categorization variable(s) used in your manuscript and explain why they were used. Please note that such variables should not be used as proxies for other socially constructed/relevant variables (for example, race or ethnicity should not be used as a proxy for socioeconomic status). Provide clear definitions of the relevant terms used, how they were provided (by the participants/respondents, the researchers, or third parties), and the method(s) used to classify people into the different categories (e.g. self-report, census or administrative data, social media data, etc.) Please provide details about how you controlled for confounding variables in your analyses.*
Population characteristics	*Describe the covariate-relevant population characteristics of the human research participants (e.g. age, genotypic information, past and current diagnosis and treatment categories). If you filled out the behavioural & social sciences study design questions and have nothing to add here, write "See above."*
Recruitment	*Describe how participants were recruited. Outline any potential self-selection bias or other biases that may be present and how these are likely to impact results.*
Ethics oversight	*Identify the organization(s) that approved the study protocol.*

Note that full information on the approval of the study protocol must also be provided in the manuscript.

Field-specific reporting

Please select the one below that is the best fit for your research. If you are not sure, read the appropriate sections before making your selection.

[x] Life sciences   [ ] Behavioural & social sciences   [ ] Ecological, evolutionary & environmental sciences

For a reference copy of the document with all sections, see nature.com/documents/nr-reporting-summary-flat.pdf

Life sciences study design

All studies must disclose on these points even when the disclosure is negative.

Sample size	Sample sizes were determined by case availability in the participating cohorts and clinical trials; no statistical method was used to predetermine sample size. For the overall benchmark dataset, we included n=3,708 early-stage breast cancers (n=1,938 TNBC; n=1,770 HER2+), spanning both resections and pre-treatment biopsies from multiple cohorts/trials.
Data exclusions	Exclusion criteria were cohort-specific and described in the Materials section. In FinHER, patients with incomplete clinical data were excluded. For GBG biopsy cohorts, all 1,928 patients were used for pCR analyses, while survival analyses used smaller evaluable subsets; differences were due to unavailable outcome data and missing clinicopathological variables required for multivariable adjustment. For SCDC, the cohort was not used for survival analyses because of limited events and high censoring and was used only for correlation and pCR prediction.
Replication	This work is a retrospective computational benchmark. Reproducibility was supported by (i) a standardized, container-based evaluation on sequestered test data via the Grand Challenge platform, and (ii) public release of training data subsets and the code used for evaluation metrics and baseline pipelines (see Data/Code Availability). The main analyses are deterministic re-runs of the same code on the same datasets; no wet-lab replication experiments were part of the main study.
Randomization	Randomization was not applicable to this retrospective validation/benchmark study; participants were not allocated to groups by the investigators in this work.
Blinding	For the model benchmark, developers had no direct access to the sequestered test datasets during model development, as evaluation was performed on the Grand Challenge platform. Outcome assessment followed a pre-specified statistical analysis plan.

Reporting for specific materials, systems and methods

We require information from authors about some types of materials, experimental systems and methods used in many studies. Here, indicate whether each material, system or method listed is relevant to your study. If you are not sure if a list item applies to your research, read the appropriate section before selecting a response.

Materials & experimental systems

n/a	Involved in the study	
☐	☒	Antibodies
☒	☐	Eukaryotic cell lines
☒	☐	Palaeontology and archaeology
☒	☐	Animals and other organisms
☐	☒	Clinical data
☒	☐	Dual use research of concern
☒	☐	Plants

Methods

n/a	Involved in the study	
☒	☐	ChIP-seq
☒	☐	Flow cytometry
☒	☐	MRI-based neuroimaging

Antibodies

Antibodies used	Re-staining/IHC subset: primary antibodies were used as a mix of CD3 (rabbit monoclonal antibody, clone SP7, Thermo Fisher Scientific, Fremont, CA; 1:100) and CD79a (clone JCB117, Agilent, Santa Clara, CA; 1:800). Detection used Envision Flex HRP DAKO DM 842 (anti-mouse/rabbit), with DAB color development and hematoxylin counterstain.
Validation	The manuscript/supplement does not report additional in-study validation experiments. The CD3 (SP7) and CD79a (JCB117) antibodies are standard, commercially supplied IHC reagents used here to generate lymphocyte-sensitive staining for the re-staining reference subset.

Clinical data

Policy information about clinical studies
All manuscripts should comply with the ICMJE guidelines for publication of clinical research and a completed CONSORT checklist must be included with all submissions.

Clinical trial registration	This study is a retrospective secondary analysis/benchmark based on previously collected cohorts and clinical trials and was not separately registered. Registration details for contributing trials are available in their original trial publications.
Study protocol	No new interventional trial protocol was created for this retrospective benchmark study. The underlying cohorts and randomized trials were defined and reported previously, and this study followed a pre-specified statistical analysis plan for outcome assessment.
Data collection	We analyzed digitized H&E whole-slide images and linked clinicopatholoical/outcome data from multiple institutions and countries, including routine clinical practice cohorts and phase 3 clinical trial material, comprising both surgical resections and pre-treatment core-needle biopsies. For survival analyses on resections, the Radboudumc surgical cohorts included TNBC resections collected between 2006–2014 (RUMC-SURVTNBC) and HER2+ resections collected between 2006–2013 (RUMC-SURVHER2+); the FinHER trial contributed scanned resections. For biopsy cohorts treated with neoadjuvant chemotherapy, we included GBG trials (GeparSixto/

	GeparSepto/GeparOcto), DIGITILS, NKI, and SCDC. Slides were stained and scanned at the contributing sites using site-specific scanners and settings, as described in the Materials section.
Outcomes	Primary outcomes were defined per analysis component. (i) Computer-vision performance: tissue segmentation accuracy (Dice score for invasive tumor and tumor-associated stroma) and lymphocyte/plasma-cell detection performance (FROC score). (ii) Agreement with visual scoring: Spearman rank correlation between computational TIL scores (cTILs) and pathologist visual stromal TIL scores (vTILs). (iii) Prediction of response to neoadjuvant chemotherapy on biopsies: pCR treated as a binary endpoint and assessed using ROC/AUC; pCR definitions followed cohort standards.. (iv) Prognostic value: association with DFI and OS assessed using Cox regression (univariable and multivariable) and concordance (C-index), with cohort-specific analyses.

Plants

Seed stocks	*Report on the source of all seed stocks or other plant material used. If applicable, state the seed stock centre and catalogue number. If plant specimens were collected from the field, describe the collection location, date and sampling procedures.*
Novel plant genotypes	*Describe the methods by which all novel plant genotypes were produced. This includes those generated by transgenic approaches, gene editing, chemical/radiation-based mutagenesis and hybridization. For transgenic lines, describe the transformation method, the number of independent lines analyzed and the generation upon which experiments were performed. For gene-edited lines, describe the editor used, the endogenous sequence targeted for editing, the targeting guide RNA sequence (if applicable) and how the editor was applied.*
Authentication	*Describe any authentication procedures for each seed stock used or novel genotype generated. Describe any experiments used to assess the effect of a mutation and, where applicable, how potential secondary effects (e.g. second site T-DNA insertions, mosiacism, off-target gene editing) were examined.*
