# Supplementary material for: Analysis of computational tumor-infiltrating lymphocytes in breast cancer from the results of the TIGER challenge
Source: Nat Commun. 2026 May 15;17:6480. doi: 10.1038/s41467-026-72956-x (PMC13377072; doi:10.1038/s41467-026-72956-x)
Supplement: Supplementary file 3 — Transparent Peer Review file [file 41467_2026_72956_MOESM3_ESM.pdf]

nature portfolio

Peer Review File

Analysis of computational tumor-infiltrating lymphocytes in breast cancer from the results of the TIGER challenge

Corresponding Author: Dr Francesco Ciompi

This manuscript has been previously reviewed at another journal. This document only contains information relating to versions considered at Nature Communications.

This file contains all reviewer reports in order by version, followed by all author rebuttals in order by version.

Version 0:

Reviewer comments:

Reviewer #3

(Remarks to the Author)
The authors have addressed my comments.

(Remarks on code availability)

Reviewer #4

(Remarks to the Author)
This is an impressive piece of work. The authors have brought together a large, multi-centric dataset and used it to address a clinically relevant problem. The challenge-based framework is innovative and sets a high standard for reproducible evaluation of AI biomarkers in pathology.

All Reviewer 1's comments have been directly addressed with manuscript changes, additional analyses, and justified explanations. Overall, this study represents a substantial and credible contribution to the field of computational pathology.

(Remarks on code availability)

Open Access This Peer Review File is licensed under a Creative Commons Attribution 4.0 International License, which permits use, sharing, adaptation, distribution and reproduction in any medium or format, as long as you give appropriate credit to the original author(s) and the source, provide a link to the Creative Commons license, and indicate if changes were made.
In cases where reviewers are anonymous, credit should be given to 'Anonymous Referee' and the source.
The images or other third party material in this Peer Review File are included in the article's Creative Commons license, unless indicated otherwise in a credit line to the material. If material is not included in the article's Creative Commons license and your intended use is not permitted by statutory regulation or exceeds the permitted use, you will need to obtain permission directly from the copyright holder.
To view a copy of this license, visit https://creativecommons.org/licenses/by/4.0/

Response to referees

All reviewers agree that the manuscript is now ready for acceptance as we have addressed all previous comments successfully, as indicated by their comments below.

We thank all reviewers for handling our manuscript and their feedback, which was invaluable to increase the quality of our paper.

Together with this letter, we are submitting the final version of our manuscript for final publication.

In our revised manuscript, we have indicated in blue color the textual changes that we made to align the structure of our final version with editorial requirements.

We also aligned the terminology of the performance metrics used in the challenge with its description in the Supplementary material, where we renamed Uno's C-index with Uno's (time-dependent) AUC, to be aligned with the terminology used in the main manuscript and in the evaluation code publicly released with the challenge. Here, we did not mark our changes in blue as we understand that the Supplementary material file will not be further edited but instead shared "as is".

Reviewer #3 (Remarks to the Author):

The authors have addressed my comments.

Reviewer #4 (Remarks to the Author):

This is an impressive piece of work. The authors have brought together a large, multi-centric dataset and used it to address a clinically relevant problem. The challenge-based framework is innovative and sets a high standard for reproducible evaluation of AI biomarkers in pathology.

All Reviewer 1's comments have been directly addressed with manuscript changes, additional analyses, and justified explanations. Overall, this study represents a substantial and credible contribution to the field of computational pathology.
